# Actual and Preferred Place of Death of Home-Dwelling Patients in Four European Countries: Making Sense of Quality Indicators

**DOI:** 10.1371/journal.pone.0093762

**Published:** 2014-04-08

**Authors:** Maaike L. De Roo, Guido Miccinesi, Bregje D. Onwuteaka-Philipsen, Nele Van Den Noortgate, Lieve Van den Block, Andrea Bonacchi, Gé A. Donker, Jose E. Lozano Alonso, Sarah Moreels, Luc Deliens, Anneke L. Francke, and on behalf of EURO IMPACT

**Affiliations:** 1 Department of Public and Occupational Health, Expertise Center of Palliative Care, VU University medical center, EMGO Institute for Health and Care Research, Amsterdam, the Netherlands; 2 Clinical and Descriptive Epidemiology Unit, Cancer Prevention and Research Institute (L’Istituto per lo Studio e la Prevenzione Oncologica, ISPO), Florence, Italy; 3 Department of Geriatrics, Ghent University Hospital, Ghent, Belgium; 4 End-of-life Care Research Group Vrije Universiteit Brussel (VUB) and Ghent University, Vrije Universiteit Brussel (VUB), Brussels, Belgium; 5 NIVEL, Netherlands Institute for Health Services Research, Utrecht, the Netherlands; 6 Public Health Directorate General, Regional Ministry of Health, Government of Castilla y León, Valladolid, Spain; 7 Health Services Research, Scientific Institute of Public Health, Public Health and Surveillance (WIV-ISP, Wetenschappelijk Instituut Volksgezondheid, Institut Scientifique de Santé Publique), Brussels, Belgium; Davidoff Center, Israel

## Abstract

**Background:**

Dying at home and dying at the preferred place of death are advocated to be desirable outcomes of palliative care. More insight is needed in their usefulness as quality indicators. Our objective is to describe whether “the percentage of patients dying at home” and “the percentage of patients who died in their place of preference” are feasible and informative quality indicators.

**Methods and Findings:**

A mortality follow-back study was conducted, based on data recorded by representative GP networks regarding home-dwelling patients who died non-suddenly in Belgium (n = 1036), the Netherlands (n = 512), Italy (n = 1639) or Spain (n = 565). “The percentage of patients dying at home” ranged between 35.3% (Belgium) and 50.6% (the Netherlands) in the four countries, while “the percentage of patients dying at their preferred place of death” ranged between 67.8% (Italy) and 86.0% (Spain). Both indicators were strongly associated with palliative care provision by the GP (odds ratios of 1.55–13.23 and 2.30–6.63, respectively). The quality indicator concerning the preferred place of death offers a broader view than the indicator concerning home deaths, as it takes into account all preferences met in all locations. However, GPs did not know the preferences for place of death in 39.6% (the Netherlands) to 70.3% (Italy), whereas the actual place of death was known in almost all cases.

**Conclusion:**

GPs know their patients’ actual place of death, making the percentage of home deaths a feasible indicator for collection by GPs. However, patients’ preferred place of death was often unknown to the GP. We therefore recommend using information from relatives as long as information from GPs on the preferred place of death is lacking. Timely communication about the place where patients want to be cared for at the end of life remains a challenge for GPs.

## Introduction

The majority of people, both the general public and terminally ill patients, prefer to die at home [1]–[4]. Therefore, the place where people die has received a great deal of interest in the last few decades and is now an extensively studied subject worldwide [5]–[11]. The proportion of people dying at home ranges from 12% to 60% [4], [6]–[10], [12]–[14]. Traditionally, palliative care professionals have tried to ensure that people are cared for at home until the end of life [15]–[17], considering dying at home as more natural [18]. Home deaths may be considered as an outcome of high quality palliative care. The view of the home as the optimal place to die has been challenged, with the establishment of palliative care in hospitals, nursing homes, hospices and other in-patient facilities [17], [19]. Interpreting the proportion of home deaths as an indicator of high-quality palliative care implies that people who were not able to die at home only received second-best care [20]–[22]. Not being able to die at home could therefore be seen as a failure in the care given to these patients, even if the patient was actually admitted to e.g. a hospital or hospice for perfectly valid reasons and in accordance with the patient’s wishes [15], [20].

Looking at whether patients die at their preferred place may therefore do more justice to the diversity of characteristics and preferences of patients. Some authors have therefore stated that ensuring death occurs in the preferred place is a more appropriate reflection of the quality than the proportion of home deaths [2], [10], [23]–[25]. Their main criticisms of home deaths as a quality indicator are that this implies a home death is optimal for the patients whereas it is not always realistic [5], [8], [10], [15], [17], [22], [26]–[29], due to the high burden on informal caregivers, the inadequate quality and quantity of resources in the home situation and the unrelieved suffering. On top of that and partly for the same reasons, a minority of patients do prefer other care locations in contrast to the majority of patients who prefer to die at home [3], [26], [30]–[33]. Therefore, whether patients die at their preferred place has only recently started to receive attention [1], [31], [32], [34], [35]. Studies show that people die at the preferred place of death in 29% to 94% of cases [1], [2], [26].

The actual place where people die and whether people die at their preferred place are often mentioned in studies aiming at improving care at the end of life, suggesting that they could function as indicators of the quality of palliative care [1], [24], [36]–[40]. Quality indicators are explicitly defined, measurable items referring to the outcomes, processes or structure of care [41], [42]. A recent systematic review [43] revealed over 300 quality indicators developed for palliative care; this included indicators focusing on the place of death and preferred place of death, but to our knowledge their actual function as indicators of the quality of care has never been studied in detail [15]. Considering the growing attention paid to quality indicators in recent years [43], [44], studying the actual place of death and preferred place of death from a quality indicator perspective could provide useful new insights.

In this paper, we want to ascertain whether the quality indicators ‘the percentage of patients dying at home’ and ‘the percentage of patients who died in their place of preference’ are feasible and informative quality indicators. This paper aims to answer the following research questions in a population of patients who died non-suddenly and who were living at home in the last month of life in Belgium, the Netherlands, Italy and Spain:

- What are the scores of the two quality indicators for home-dwelling patients with a non-sudden death in Belgium, the Netherlands, Italy and Spain?
- Are these quality indicators feasible in terms of the number of missing values when derived from the data of representative general practitioner (GP) networks?
- Are quality differences between countries revealed in these indicator scores? What kind of information do the two quality indicators give us in terms of measured quality? Do they overlap, or should they be used in combination?
- Are the expected differences in quality indicator scores between countries related to differences in care characteristics (adjusting for differences in patient characteristics)? If so, this means that influencing these care characteristics may lead to more patient-centred care, reflected in higher indicator scores, meaning more people would die at home and/or at their preferred place.

## Methods

### Study Design

Data came from the European Sentinel GP Networks Monitoring End-of-Life Care (EURO SENTI-MELC) study, a mortality follow-back study on monitoring end-of-life care in four European countries, namely Belgium, the Netherlands, Spain and Italy. For this study, we used data from the nationally representative GP networks collected in 2009 (all countries except Spain), 2010 (all four countries) and 2011 (Spain only). The GP sentinel networks cover 1.8% and 0.8% of the Belgian and Dutch national populations respectively [45]–[47]. The Spanish sentinel network represents 3.5% of the patient population in the Castilla and León region (in the northwest) and 2.2% in the Valencia region (in the east) [47], [48]. The Italian data came from a new GP network set up for this study [49] and were collected from nine of the 146 health districts, covering about 4% of the patient population [47].

### Study Population

**Figure 1:**
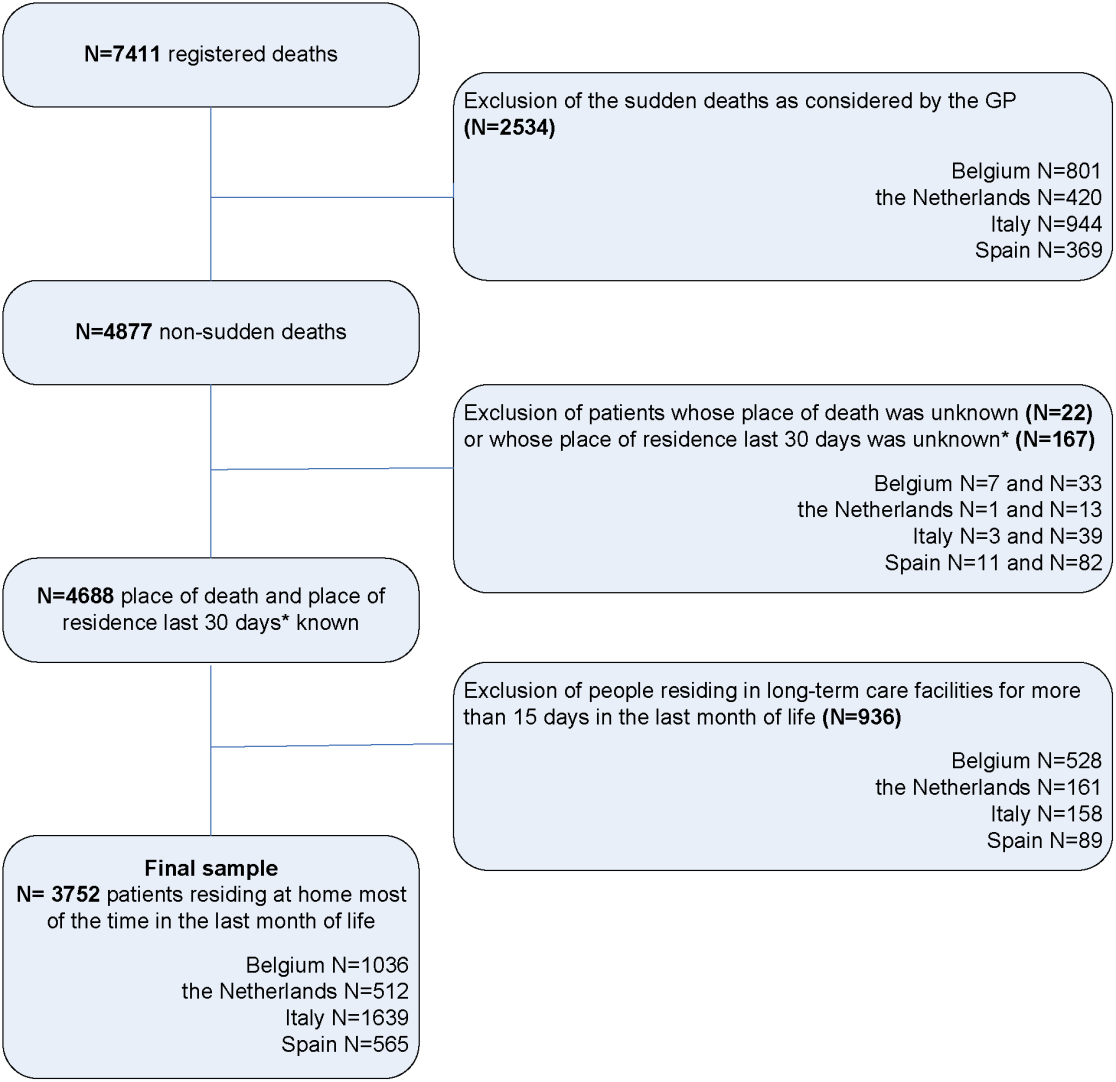
Flowchart of the sample selection. *We excluded patients if place of residence was known for ≤15 days in the last month of life OR if place of residence was known for <30 days and a transition took place during this period.

The recorded data were analysed of deceased adult patients (aged 18 and above), who were part of a GP’s practice and had died non-suddenly according to their GP. Since this study examines the care delivered at the end of life, the data of people who died suddenly and unexpectedly according to their GP were excluded, leaving a population that was eligible for palliative care [45]. Furthermore, the data of deceased people who had been living in long-term care facilities (nursing homes, residential homes or care homes) for more than 15 days in the last month of life were excluded in all four countries. This choice was made since we were primarily interested in the place of death and preferred place of death of people mainly living at home, and also to enhance comparability of the datasets of the four countries involved since the Dutch SENTI-MELC data set did not include nursing-home residents (in Dutch nursing homes, elderly-care physicians have the medical responsibility rather than GPs [50]). Figure 1 shows a flowchart of the selected sample.

### Selected Quality Indicators

For the selection of the quality indicators, we used a list of 326 quality indicators for palliative care found in a recent systematic review [43]. Four of these 326 indicators were related to the actual place of death and eight indicators concerned dying at the preferred place of care. From these twelve indicators, we selected two indicators that we could calculate with the existing EURO SENTI-MELC dataset. The first quality indicator selected, ‘the percentage of patients dying at home’, comes from a set of quality indicators developed in Italy for palliative home care [40]. The indicator is calculated using ‘the number of patients dying at home’ as the numerator and ‘the total number of patients’ as the denominator. The performance standard specified by the developers is that at least 95% of the patients receiving home palliative care should die at home. The second quality indicator selected concerns ‘the percentage of patients who died in the location of their preference’. This quality indicator was found in two indicator sets that were developed for a wider range of settings [24], [38]. In one of the sets [38] the indicator was calculated using ‘the number of relatives who indicate that the patient died in the location of his/her preference’ as the numerator and ‘the total number of relatives for whom this quality indicator was measured’ as the denominator. We used the GP’s knowledge of the patient's preferred place of death to calculate this indicator.

### Data Collection

The data needed for the calculation of these two quality indicators were taken from the data of the EURO SENTI-MELC study in which GPs recorded the characteristics of recently deceased patients on weekly basis using a standardised questionnaire. Recall bias was minimised by requiring registration to be no more than one week after the GP had been informed of the patient’s death [47]. In the questionnaire, GPs were asked about the actual place of death [at home or living with family, in a care home (Belgium and Italy)/elderly home (the Netherlands and Spain), in hospital, in a palliative care unit/hospice, or elsewhere (namely); dichotomised into ‘at home’ (i.e. at home or living with family) vs. ‘not at home’].

In addition, the patient’s preference regarding place of death was asked in the question ‘Were you informed (verbally or in writing) of the patient’s preference regarding place of death?’. If the answer to this question was ‘yes’, the GP was then asked ‘Where did this patient prefer to die?’ and could choose from these options: at home or living with family, in a care home (Belgium and Italy)/elderly home (Netherlands and Spain), in hospital, in a palliative care unit/hospice or elsewhere (namely). The questionnaire also included the following questions:

- The provision of palliative care by the GP, as judged by the GPs themselves [no; yes, but not until death; yes, until death; dichotomized into ‘yes’ and ‘no’];
- The importance of care goals in the second to fourth week before the patient died, as judged by the GPs themselves: treatment aimed at cure, treatment aimed at prolonging life and treatment aimed at palliation, rated on a five-point Likert scale (1 ‘not at all important’ to 5 ‘very important’). These scores were dichotomized into the categories ‘important to very important’ (scores of 4 and 5) and ‘not so important’ (scores of less than 4).

### Informed Consent and Patient Anonymity

After being informed of the objectives and procedures of the study, participating GPs gave written informed consent at the beginning of each registration year. Strict procedures regarding patient anonymity were employed during data collection and entry; every patient received an anonymous reference code from their GP and any identifying patient and GP data (such as date of birth, postcode and GP identification number) were replaced with aggregate categories or anonymous codes.

### Ethical Approval

The protocol of this study was approved by the Ethical Review Board of Brussels University Hospital of the Vrije Universiteit Brussel (2004), Belgium, and the Local Ethical Committee, ‘Comitato Etico della Azienda U.S.L. n. 9 di Grosseto’ (2008), Tuscany, Italy. In the Netherlands and Spain, no ethical approval is required for the posthumous collection of anonymous patient data.

### Statistical Analysis

We calculated the quality indicator ‘the percentage of patients dying at home’ from the question concerning the place of death. The quality indicator ‘the percentage of patients who died in the place of their preference’ was calculated based on the combined information concerning actual and preferred place of death. Descriptive statistics were used to describe the study population and the quality indicator scores.

To enable a valid comparison between countries in quality indicator scores, the quality indicator scores were standardised for patients’ gender, age at death, cause of death and diagnosis of dementia, using the distribution observed in the study population as a whole as the reference distribution.

Multivariable logistic regression analyses were performed to identify the care characteristics associated with dying at home and dying at the place of preference adjusting for patient characteristics. The patient characteristics used for adjustment were gender [‘male’ vs. ‘female’], age at death [‘18–64’, ‘65–84’ or ‘85 and older’], cause of death [‘cancer’ vs. ‘non-cancer’] and diagnosis of dementia [‘no’, ‘yes, mild dementia’ and ‘yes, severe dementia’]. The care characteristics analysed were ‘GP provided palliative care’ [‘yes’ vs. ‘no’] and care goals in the last 2–4 weeks of life of ‘treatment aimed at cure’, ‘treatment aimed at prolonging life’ and ‘treatment aimed at comfort/palliation’ [‘important to very important’ vs. ‘not so important’]. We performed a separate analysis per country, using a single multivariable model for each country, including the confounders (age, gender, cause of death, diagnosis of dementia) and the predictors (‘GP provided palliative care’ and the three care goals). We retained the confounders in the model regardless of their statistical significance. The analyses were performed using IBM SPSS Statistics software, Version 20.0 (IBM Corp., 2011, Armonk, NY), with a significance level α<0.05.

## Results

### Description of the Sample

Of the original 7411 red deaths, GPs considered 4877 deaths as non-sudden. Exclusion of long-term care facility residents in all four countries left a total number of 3752 deaths: 1036 for Belgium, 512 for the Netherlands, 1639 for Italy and 565 for Spain (see Figure 1). In all countries except for Italy, the majority of the patients in the samples were male (Table 1). About one quarter of the Belgian and Dutch samples were aged 85 or older, whereas this group of the very elderly comprised around 40% in Italy and Spain. Malignancy was the main cause of death in all countries, but the proportion in the Netherlands was higher (60.8% versus 40.8–48.4%). Fewer patients were diagnosed with dementia in the Netherlands than in the other three countries (7.3% versus 17.4–27.7%).

**Table 1:** Characteristics of the patients and of the care provided per country.

		BELGIUM (N = 1036)	THE NETHERLANDS (N = 512)	ITALY (N = 1639)	SPAIN (N = 565)
		N (%)	N (%)	N (%)	N (%)
PATIENT CHARACTERISTICS					
**Gender** *	Female	471 (45.6)	235 (46.4)	857 (52.3)	249 (44.6)
	Male	563 (54.4)	271 (53.6)	782 (47.7)	309 (55.4)
**Age at death** †	18–64	199 (19.4)	113 (22.1)	217 (13.2)	64 (11.3)
	65–84	559 (54.4)	280 (54.7)	779 (47.5)	268 (47.4)
	85 and older	269 (26.2)	119 (23.2)	643 (39.2)	233 (41.2)
**Cause of death** ‡	Cancer	501 (48.4)	310 (60.8)	767 (47.9)	226 (40.8)
	Cardiovascular diseases (except stroke)	135 (13.0)	62 (12.2)	327 (20.4)	105 (19.0)
	Respiratory diseases	95 (9.2)	42 (8.2)	117 (7.3)	59 (10.6)
	Neurologic diseases	47 (4.5)	14 (2.7)	89 (5.6)	29 (5.2)
	CVA - stroke	57 (5.5)	18 (3.5)	149 (9.3)	47 (8.5)
	Other	200 (19.3)	64 (12.5)	151 (9.4)	88 (15.9)
**Diagnosed dementia** §	No	844 (82.6)	458 (92.7)	1183 (73.1)	401 (72.4)
	Yes, mild dementia	102 (10.0)	22 (4.5)	228 (14.1)	79 (14.3)
	Yes, severe dementia	76 (7.4)	14 (2.8)	207 (12.8)	74 (13.4)
**CARE CHARACTERISTICS**					
**GP provided palliative care** ||	No	573 (55.4)	264 (39.7)	725 (44.3)	207 (38.8)
	Yes	462 (44.6)	299 (60.3)	910 (55.7)	326 (61.2)
**Cure is a (very) important care goal in week 2–4 before death** ¶		227 (24.0)	60 (14.2)	230 (16.5)	85 (19.5)
**Prolonging life is a (very) important care goal in week 2–4** **before death** **		304 (31.9)	90 (21.5)	558 (39.3)	112 (26.7)
**Palliation is a (very) important care goal in week 2–4** **before death** ††		647 (68.5)	374 (87.8)	781 (60.2)	304 (67.1)

*Missing values: Belgium N = 2, the Netherlands N = 6, Italy no missing values, Spain N = 7.

† Missing values: Belgium N = 9, the Netherlands, Italy and Spain no missing values.

‡ Missing values: Belgium N = 1, the Netherlands N = 2, Italy N = 39, Spain N = 11.

§ Missing values: Belgium N = 14, the Netherlands N = 18, Italy N = 21, Spain N = 11.

|| Missing values: Belgium N = 1, the Netherlands N = 16, Italy N = 4, Spain N = 32.

¶ Missing values: Belgium N = 90, the Netherlands N = 88, Italy N = 244, Spain N = 132.

**Missing values: Belgium N = 84, the Netherlands N = 94, Italy N = 219, Spain N = 146.

†† Missing values: Belgium N = 91, the Netherlands N = 86, Italy N = 342, Spain N = 112.

In all countries except for Belgium, the majority of patients received palliative care from their GP (Table 1). Palliation was considered an important care goal in the last 2–4 weeks of life for the majority of the patients in all countries. Cure was still an important care goal in 14.2–24.0% of patients and prolonging life in 21.5–39.3% (Table 1).

### Quality Indicator Scores per Country

Belgium had the lowest scores on the standardised quality indicator ‘the percentage of patients dying at home’: in Belgium, only 35.3% of the sample of GPs’ patients living at home and with a non-sudden death died at home. Home deaths accounted for 49.1–50.6% in the samples in the other three countries (Table 2).

**Table 2:** Observed and standardised quality indicator (QI) scores per country.

	BELGIUM (N = 1036)	THE NETHERLANDS (N = 512)	ITALY (N = 1639)	SPAIN (N = 565)
**% of patients dying at home**	34.7%	52.5%	50.9%	51.3%
**Standardised % of patients dying at home** *	35.3%	50.6%	49.1%	50.5%
N unanswered questions†	7	1	3	11
**% of patients who died in the location of their preference** ‡	72.3%	83.2%	69.7%	87.9%
**Standardised % of patients who died in the location of their preference** *	72.6%	75.4%	67.8%	86.0%
N unanswered or inconsistently answered questions	7	10	7	66
N (%) preference unknown by GP	592 (57.5%)	199 (39.6%)	1147 (70.3%)	334 (66.9%)

*These percentages have been standardised for gender, age, cause of death and diagnosis of dementia.

† These patients were excluded from our study (see Figure 1).

‡ This quality indicator was only calculated when preference was known: Belgium (n = 437), the Netherlands (n = 303), Italy (n = 485) and Spain (n = 165).

Italy had the lowest scores for the standardised quality indicator ‘the percentage of patients who died at their preferred place of death’: in Italy, 67.8% of the GPs’ patients who lived at home and died non-suddenly died at the preferred place, while this percentage was highest in Spain (86.0%) (Table 2). These quality indicator scores standardised for gender, age, cause of death and diagnosis of dementia, differed slightly from the crude, observed percentages, by 0.3% to 7.8% (see Table 2).

### Feasibility of Collecting the Necessary Data for the Quality Indicators

The quality indicator concerning the actual place of death had very few missing values (Table 2). The number of cases where the questions were not answered or inconsistently answered was also low for the quality indicator concerning the preferred place of death (Table 2). On the other hand, high numbers of unknown preferences were seen for this indicator: from 39.6% in the Netherlands to 70.3% in Italy (Table 2). The proportion of cases where the preferences were unknown differed substantially between home deaths and deaths outside the home (p<0.001 in all four countries): the percentage of unknown preferences was higher for deaths outside the home, and this was the case for all four countries (Table 3).

**Table 3:** Comparison of the two quality indicators per country.

	BELGIUM (N = 1036)		THE NETHERLANDS (N = 512)		ITALY (N = 1639)		SPAIN (N = 565)	
	At home	Not at home	At home	Not at home	At home	Not at home	At home	Not at home
	N (%)*	N (%)†	N (%)*	N (%)†	N (%)*	N (%)†	N (%)*	N (%)†
**Preference met**	254 (70.6)	62 (9.2)	215 (79.9)	37 (15.2)	318 (38.1)	20 (2.5)	136 (46.9)	9 (3.3)
**Preference not met**	5 (1.4)	116 (17.2)	7 (2.6)	44 (18.1)	2 (0.2)	145 (18.0)	1 (0.3)	19 (6.9)
**Preference unknown** ‡	101 (28.1)	498 (73.7)	47 (17.5)	162 (66.7)	515 (61.7)	639 (79.5)	153 (52.8)	247 (89.8)

*Percentages are the percentage of deaths at home.

† Percentages are the percentage of deaths not at home.

‡ Including unanswered and inconsistently answered questions.

### Comparison of the Outcomes of the Two Quality Indicators

A fair, simple comparison of the outcomes of two indicators is impossible, firstly due to the high percentage of missing information for the preferred place of death and secondly due to the fact that the proportion of missing values varies between countries and place of death (from 17.5% missing for patients in the Netherlands who died at home to 89.8% missing for patients in Spain who did not die at home, Table 3). For 71% of the patients in Belgium and 80% of the patients in the Netherlands who died at home, this was in accordance with their preferences known by the GP (Table 3). In Italy and Spain, these percentages were lower (38% and 47% respectively). Some people did not die at home but did die in the location of their preference, from 3% (Italy) to 15% (the Netherlands). The reverse (people who died at home when that was not the preferred place) occurred too.

### Care Characteristics Associated with Quality Indicators

Receiving palliative care from the GP is positively associated with dying at home (Table 4). This association is greatest in Belgium and the Netherlands (OR of 8.37 and 13.23 respectively). If cure is an important care goal in the last 2–4 weeks of life, people are less likely to die at home. This association is only significant in Belgium and Spain (OR of 0.57 and 0.48 respectively). If prolonging life is an important care goal in the last weeks of life, people are less likely to die at home. This association was only significant in Italy and Spain (OR 0.75 and 0.41 respectively). Palliation as an important care goal does not seem to have a consistent association with the place of death.

**Table 4:** Associations of care characteristics with the two quality indicators per country.

	BELGIUM		THE NETHERLANDS		ITALY		SPAIN	
	Home death	Preference met	Home death	Preference met	Home death	Preference met	Home death	Preference met
	(N = 1036)	(N = 437)	(N = 512)	(N = 303)	(N = 1639)	(N = 485)	(N = 565)	(N = 165)
	OR (95% CI)	OR (95% CI)	OR (95% CI)	OR (95% CI)	OR (95% CI)	OR (95% CI)	OR (95% CI)	OR (95% CI)
**GP provided palliative care** †	8.37 (5.7–12.2)**	4.14 (2.4–7.1)**	13.23 (7.2–24.4)**	6.63 (2.6–17.1)**	1.55 (1.2–2.0)*	2.30 (1.4–3.9)*	3.80 (2.3–6.3)**	3.87 (0.9–16.8)
**Cure is a (very) important care goal in week 2–4 before death** ‡	**0.57 (0.3–1.0)**	0.80 (0.4–1.8)	0.43 (0.2–1.2)	1.42 (0.2–8.1)	0.81 (0.6–1.2)	0.88 (0.4–1.8)	**0.48 (0.2–0.9)**	0.39 (0.1–2.3)
**Prolonging life is a (very) important care goal in week 2–4 before** **death** ‡	0.75 (0.5–1.1)	0.78 (0.4–1.5)	0.56 (0.3–1.1)	0.52 (0.2–1.6)	**0.75 (0.6–1.0)**	0.65 (0.4–1.8)	0.41 (0.2–0.7)*	0.58 (0.1–3.3)
**Palliation is a (very) important care goal in week 2–4 before death** ‡	0.93 (0.6–1.4)	0.94 (0.5–1.8)	1.01 (0.4–2.4)	0.89 (0.2–3.8)	1.03 (0.8–1.3)	1.25 (0.7–2.1)	0.90 (0.5–1.5)	0.32 (0.1–1.7)

In this multivariable regression analyses, we corrected for gender, age at death, cause of death, diagnosis of dementia.

Odds ratios marked in bold are significant p<0.05.

Odds ratios marked in bold, with 1*are significant p<0.01.

Odds ratios marked in bold, with 2 **are significant p<0.001.

† Reference category =  no palliative care provided by the GP.

‡ Reference category =  care goal considered as not so important.

Dying at the place of preference is also positively associated with receiving palliative care from the GP in all countries, except for Spain (Table 4). The associations of other care characteristics with dying at the preferred place are not statistically significant.

## Discussion

This is the first cross-national study to compare two quality indicators concerning the actual and preferred place of death for patients living at home who died non-suddenly. The percentage of home deaths varied between 35.3% (Belgium) and 50.6% (the Netherlands). Of patients whose preference for place of death was known, 67.8% (Italy) to 86.0% (Spain) died in the location of their preference. The quality indicator concerning the percentage of home deaths is easy to collect and measurement by GPs is feasible. However, the feasibility of the indicator concerning dying at the preferred place of death is hampered due to the high percentage of patients’ preferences unknown by the GP (39.6%–70.3%). Despite the high percentage of unknown preferences, the results indicate that there is a strong overlap between home deaths and deaths in the preferred location. Quality indicator scores are related to care characteristics: patients receiving palliative care from the GP were more likely to die at home and to die at the place of preference; and people were less likely to die at home if ‘cure’ or ‘prolonging life’ was an important care goal in the last 2–4 weeks of life.

Regarding the feasibility of collecting these data with the help of GPs, the quality indicator concerning home deaths had very few missing values, which shows that calculating this quality indicator with data gathered by GPs is feasible. The number of unanswered or inconsistently answered questions was also low for the quality indicator concerning the preferred place of death. However, high numbers of unknown preferences (39.7–69.8%) were seen for this indicator. Other studies have found unknown preference rates varying between 12% and 64% [1], [2], [12], [30], [31], [46], [51]. The proportion of unknown preferences was highest in the group of non-home deaths in all four countries, which is consistent with the findings of previous GP sentinel network studies [30], [52]. Exploring patients’ preferences may be a challenging process, because both the GP and the patient have to recognise the approaching end of life and have to be willing to talk about this subject [20], [21]. In addition, some patients might not have a strong or pronounced preference and recording a definitive answer might be difficult. Patients also differ in the ability or willingness to express their preferences: culturally-related inhibitions preventing patients from talking openly about death or a low level of educational might hamper timely discussion [2], [20], [27].

The indicator for the actual place of death has a defined performance standard of 95%, meaning that at least 95% of the patients receiving home palliative care should die at home [40]. One could argue that applying this performance standard to our data set is not realistic, since not all the patients in the data set received home palliative care, in contrast to the original indicator set. Alternatively, in the absence of a well-defined performance standard we can apply the ‘best-practice norm’ principle: take a look at which country scores best and recommend this score as a target that other countries should aim for in future. In this study, one could therefore 51% as the minimum for the proportion of home deaths as a best-practice norm (the highest score, achieved in the Netherlands) and a minimum of 86% of patients dying at the preferred place if the preferred place was known by the GP (the highest score, achieved in Spain). This could be a way to overcome the absence of a performance standard, using a relative rather than an absolute norm as a threshold value for the quality of care.

We also saw that there is a strong overlap between dying at home and dying in the preferred location, found in all countries. Taking into account the unknown preferences, where we do not know if the preference was met, we can be sure that the majority of Belgian and Dutch patients (71% and 80% respectively) died at home according to their wishes, whereas this was only the case in a minority of Italian and Spanish patients (38% and 47% respectively). Of the people who did not die at home, 3% to 15% still died in their place of preference. These patients were not included in the ‘dying at home’ quality indicator, suggesting that the indicator concerning preference covers a wider group of patients who died as preferred.

In addition, we revealed that some care characteristics were associated with the quality indicators, namely whether the GP provided palliative care and whether ‘cure’ or ‘life prolongation’ was an important treatment goal in the last two to four weeks of life. These effects are consistent with the existing literature: receiving chemotherapy in the last month of life has been associated with a reduced likelihood of a home death [11]; the provision of palliative care by the GP has been associated with an increased likelihood of home death [5], [19], [30], [53]–[57]; dying in the preferred place of death has been associated with GP involvement and GP home visits [32], [35]. The exact role of the GP in the provision of health care in general and more specifically in the provision of palliative care differs between countries. In the Netherlands, the GP has not only a high level of responsibility as a gatekeeper of referrals to hospital care and specialist care in general [47] but also plays the main role in the delivery of generalist palliative care at home [58], [59]. GPs in Spain also fulfil a gatekeeper function [47], but share the responsibility of the organisation for palliative care with home care teams [60]. Palliative care is also a shared responsibility of GPs and multidisciplinary palliative home care teams in Belgium [61] and Italy [62], [63]; in these countries, GPs are not gatekeepers in general, but they do have a coordinating role in the healthcare system since most people have a GP who they consult regularly. Although the role of GPs in the four countries differs, having the GP provide palliative care was positively associated with dying at home and dying in the preferred place of death in all four countries. This suggests that improving these specific aspects, e.g. in this case improving the provision of palliative care by the GP and improving the GP-patient communication concerning preferences at the end of life (including the preferred place of death) can improve the quality of palliative care, which may then be reflected in higher quality indicator scores.

Although quality indicators are developed to provide an overview for a care setting or country as a whole, not for individual patients, we do think that it is important to keep the perspective of individual patients in mind when thinking about realistic performance standards for these indicators. Achieving a situation in which all patients die at home or all preferences are known might not be desirable or realistic. Home deaths may be suggested as an outcome of high-quality palliative care, but might give the impression that home deaths are the golden standard while for some patients this is not the best or preferred option. It misses out small minorities of patients who died in their preferred location elsewhere or who died at home without preferring home. Hence, it might seem that the percentage of patients dying at the preferred place of death is a better indicator, as it takes into account all preferences met in all locations. However our study showed that at present it is not feasible for GPs to collect data for the indicator on preferred place of death due to the high percentage of cases where the preferences are unknown to the GP. We therefore recommend that GPs actively improve their communication with patients so that they are able to find out and comply with patients’ preferences. In cases where the GP is not aware of the patient’s preference, we recommend measuring the indicator concerning the preferred place of death via relatives, as was originally intended in the original indicator set and was found to be feasible in a first test [38]. Another option is that, in the meantime, place of death could be used as a proxy, since there is a big overlap between the two indicators.

Furthermore, we should note that for care providers who aim to monitor and improve the quality of care provided, using only one quality indicator concerning the place of the death is not sufficient. Using a wider range of quality indicators, concerning different physical, psychological and spiritual aspects of palliative care, is necessary to provide a more complete picture of the quality of care provided [21], [43], [64].

### Strengths and Limitations

This is the first cross-European study using existing data to compare the percentage of home deaths and the percentage of patients who died at their preferred place, and to assess their function as quality indicators for palliative care.

However, a limitation is that GPs themselves stated whether they had provided palliative care and we have no detailed information on what GPs considered as ‘providing palliative care’. The reported preferences were also based on the GP’s own observation and the high number of unknown preferences shows GPs did not know all the details of their patients’ preferences. A possible bias can be that the sampled patients had more contact with their GPs and were thus able to state their preference more clearly to their GPs.

## Conclusion

The quality indicator ‘the percentage of home deaths’ is easy for GPs to provide, but might give a narrow view of the quality of care, implying that home deaths are the golden standard. Hence it might seem that the quality indicator ‘dying at the preferred place’ is a better alternative, as it takes into account all preferences met in all locations. However, it is not feasible at present to have this indicator measured by GPs due to the high percentage of cases where the preferences are unknown to the GP. We therefore suggest using information from relatives as long as information from GPs on the preferred place of death is lacking. Since dying at the preferred place of death offers great potential for becoming a good quality indicator for palliative care, we recommend that GPs pay ample attention to communication at the end of life, exploring patients’ preferences, including the place of death.
